# Co-existence of the abdominal wall and thoracic endometriosis: case report and literature review

**DOI:** 10.3389/fmed.2025.1578435

**Published:** 2025-05-15

**Authors:** Bo Gao, Zhi Li, Hui Liu, Gongmin Zhou, Xiuxiu Lai

**Affiliations:** ^1^Department of Prosthodontics, Second Affiliated Hospital of Zhejiang University School of Medicine, Hangzhou, Zhejiang, China; ^2^Zhejiang Provincial Clinical Research Center for Oral Diseases and Key Laboratory of Oral Biomedical Research of Zhejiang Province, Hangzhou, Zhejiang, China; ^3^Department of Gynaecology and Obstetrics, Women’s Hospital School of Medicine, Zhejiang University, Hangzhou, Zhejiang, China; ^4^Department of Pathology, Second Affiliated Hospital of Zhejiang University School of Medicine, Hangzhou, Zhejiang, China; ^5^Geriatric Medicine Center, Department of Geriatric Medicine, Zhejiang Provincial People’s Hospital, Affiliated People’s Hospital, Hangzhou Medical College, Hangzhou, Zhejiang, China

**Keywords:** abdominal wall endometriosis, thoracic endometriosis, surgery, hormone therapy, recurrence

## Abstract

Extra-pelvic endometriosis always presents complex diagnostic challenges due to its non-specific symptoms. We describe a rare case of coexisting iatrogenic abdominal wall endometriosis (AWE) and thoracic endometriosis (TE) in a 33-year-old woman without pelvic involvement. The patient, with a history of two cesarean sections, presented with chronic abdominal pain and an asymptomatic pulmonary nodule. Both lesions were removed through surgical resection and histologic examination confirmed endometriosis. One year after the surgery, the patient experienced a recurrence of the abdominal pain and opted for hormone therapy. In this case, AWE is consistent with iatrogenic endometrial transplantation, while the thoracic lesion may result from blood transmission after cesarean section, but the specific mechanism remains to be further explored. We aimed to provide a novel insight into the multifactorial pathogenesis of extrapelvic endometriosis and multidisciplinary management.

## Introduction

Endometriosis is characterized by the presence of endometrial glands outside the uterus, affecting 6%–10% of reproductive age women and causing a huge economic burden (1–3). It is always found in the pelvis and less commonly found in extra-pelvic. The sites outside the pelvis include the chest, diaphragm, abdominal wall, urogenital tract and others organs (4–7). The clinical presentation varies widely, with chronic pain being one of the most common manifestations. For instance, diaphragmatic endometriosis may present with shoulder pain, chest pain, or upper abdominal discomfort (7, 8). Other non-specific symptoms include infertility, catamenial pneumothorax, and hemoptysis.

The extra-pelvic lesions are always difficult to diagnose correctly due to non-specific symptoms and lack of awareness. A review including 179 studies reported that the majority of patients with extra-pelvic endometriosis (84%) were found and treated by non-gynecologic clinicians (1). Previous researches revealed a considerable diagnostic delay ranging from 6.7 to 8.6 years (9), and a high proportion of patients were misdiagnosed as another physical (95.1%) or psychosocial problem (49.5%) (10). Among extra-pelvic endometriosis cases, abdominal wall endometriosis (AWE) and thoracic endometriosis (TE) appear to be relatively frequent, with reported incidences of 1%∼2% and 1.5%, respectively (11, 12). However, the simultaneous occurrence of multiple endometriosis lesions is extremely rare. Herein, we present a rare case of a young woman with a history of both AWE and TE, who achieved successful treatment by surgical resection and medication.

## Case description

In August 2022, a 33-year-old woman was referred to the Department of Gynecology due to recurrent low abdominal wall pain that had persisted for 3 months. The pain, located above the cesarean section scar, progressively worsened over time. Initially, she suspected a muscle strain resulting from exercise; however, adequate rest did not alleviate the pain. The discomfort recurred periodically and appeared to correlate with the menstruation. Her medical history included two previous Cesarean sections (7 and 2 years ago), but no prior history of endometriosis or endometriosis-related symptoms (e.g., dysmenorrhea, dyspareunia, or chronic pelvic pain).

**FIGURE 1:**
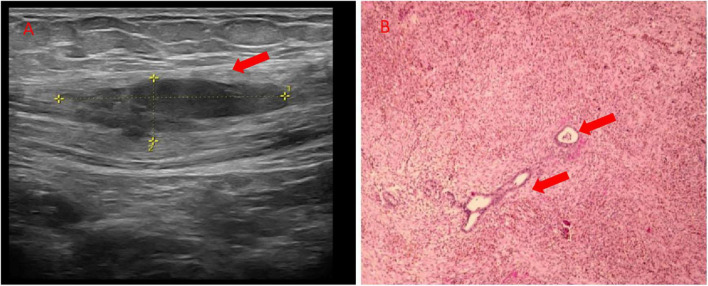
**(A)** Ultrasonography of the abdominal wall showed a 3.5 × 3.4 × 1.2 cm hypoechoic lesion with rare vascularity (arrow); **(B)** histopathological result of the abdominal mass showed endometrial glands and stroma (arrow) (200×).

Physical examination revealed a fixed, painful abdominal mass, located above the right side of the cesarean scar. Ultrasonography demonstrated a 3.5 × 3.4 × 1.2 cm hypoechoic lesion with rare vascularity in the muscular layer of abdominal wall (Figure 1A). Transvaginal ultrasound revealed normal adnexa and uterus. A diagnosis of scar endometriosis was suspected given the patient’s history of obstetric surgery and periodic pain of the mass in accordance with menstruation. Therefore, the patient underwent surgical resection of the abdominal wall mass. Intraoperative findings revealed a poorly defined, irregular, and hard nodule involving the anterior rectus sheath without deeper muscular invasion. The lesion was completely excised, and electrocautery was performed for hemostasis. No mesh reinforcement was required due to the limited defect size. Subsequently, the suspicion was confirmed by pathologic tissue examination. The biopsy specimen showed endometrial glands and stroma (Figure 1B).

**FIGURE 2:**
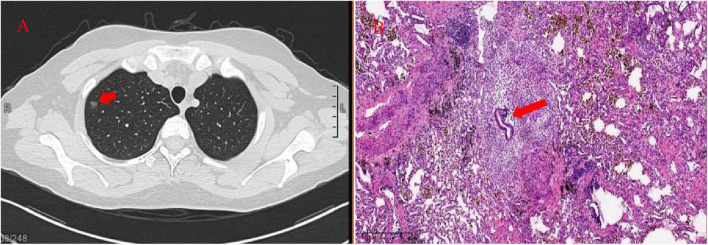
**(A)** Chest CT showed an 8 × 7 mm lung nodule with a ground-glass appearance (arrow); **(B)** histopathological result of excised lung tissues showed endometrial glands and stroma (arrow).

At the same time, chest computed tomography (CT) incidentally revealed a 5-mm ground-glass nodule in the apical segment of the right upper lobe (Image:11/67). She did not have any catamenial thoracic symptoms and denied a history of smoking. In order to exclude the possibility of pneumonia, the patient received an oral antibiotic therapy for 10 days and had a follow-up chest CT-scan 2 months later. The chest CT still showed an 8 mm solitary nodule in the same location (Figure 2A). In addition, pulmonary function tests demonstrated a mild obstructive ventilation dysfunction. Finally, the patient underwent right-sided video-assisted thoracoscopic surgery (VATS) in November 2022. A single 4-cm incision was made in the fifth intercostal space at the anterior axillary line and the upper lobe nodule was excised via wedge resection. Subsequent histopathological examination showed endometrial glands and stroma, accompanied with hemorrhage in the surrounding alveolar cavity and interstitial fibrosis (Figure 2B), revealing a diagnosis of pulmonary endometriosis. Given the lack of clinical indications for deep pelvic endometriosis, pelvic MRI was not performed.

**FIGURE 3:**
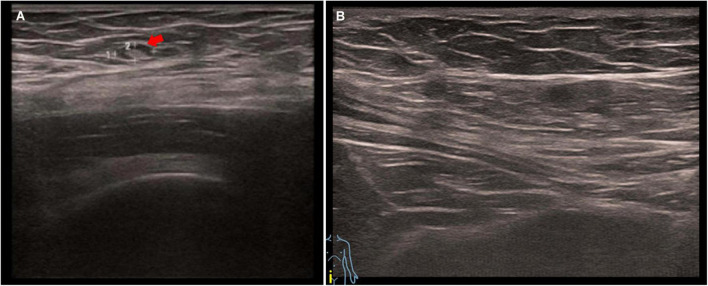
**(A)** Ultrasonography of the abdominal wall showed a 3 × 7 mm hypoechoic lesion (arrow); **(B)** the follow-up ultrasonography after 4-month treatment showed no lesion beneath the cesarean section scar.

After an 8-month follow-up period, the patient’s pulmonary lesions had exhibited no recurrence. Unfortunately, she experienced recurrent abdominal pain 1 year after the surgery, and ultrasonography showed a 3 × 7 mm hypoechoic lesion at the cesarean scar (Figure 3A). Considering lack of desire for fertility, the gynecologist recommended hormonal therapy. After 4 months of treatment with Dienogest, her abdominal pain was significantly alleviated and the follow-up ultrasonography confirmed the absorption of the lesion (Figure 3B). During the therapeutic course, the patient reported mild adverse effects including a slight weight gain and medication-induced amenorrhea, which were managed through dietary counseling and regular monitoring.

## Discussion

Endometriosis is a chronic inflammatory disorder wherein the endometrial-like tissue present outside the uterus. This abnormal tissue commonly appears on the ovaries, fallopian tubes, and the tissue lining the pelvis, although it may occasionally extend beyond the pelvic organs. Localized lesions periodically bleed, stimulate inflammatory reaction and lead to the formation of adhesions and nodules (13). Consequently, menstruation-related pain is the most common symptom. However, it is not specific to endometriosis since the pain may be caused by other non-gynecological conditions. In addition to physical symptoms, some patients suffer psychological stress, anxiety and depression, affecting the quality of life and social well-being (14).

AWE is defined as ectopic endometrial tissues found within the abdominal wall. Most cases are secondary to prior abdominal surgeries, while a small part are spontaneous lesions (15). The interval between the onset of symptoms and a related surgery ranges from 3 to 6 years (16) and the most common manifestations are abdominal mass (85.2%) and local pain (60.6%) at the surgical incision (17). The pathogenesis of AWE is complicated and the most accepted theory is the “implantation theory”. This theory suggests a type of artificial endometrial implantation to certain body parts, such as to the cesarean scar or perineal side incision during childbirth (11). Subsequently, the local lesions enlarge with each menstrual cycle, accompanied by constant pain. In this case, the diagnosis of AWE is relatively easy, for the criterion includes a fixed mass located above the cesarean scar, periodic pain and prior surgeries. Ultrasonography is a useful diagnostic tool, which can reveal hypoechoic masses near the incision (18). Other imaging tests such as CT and MRI are valuable in determine the extent of the lesions. The latter is helpful in differentiating rectus sheath hematoma from other anterior abdominal wall masses (19).

However, TE can easily be misdiagnosed because of the non-specific clinical manifestations and a low incidence. The thoracic symptoms vary according to the location of the lesion, including pneumothorax, hemothorax, hemoptysis and asymptomatic lung nodules (1), which are always coordinated to the menses. Among them, lung nodules are the least common manifestations, accounting only for 4.5%–6% (1, 20). In the current case, the patient had no typical chest symptom except for a lung nodule in the right lobe. Although she had two cesarean sections and a history of AWE, it was difficult to make a correct diagnosis of pulmonary endometriosis.

How endometrial cells reach the lung remains unknown. The pathogenesis is complex and multifactorial, and various theories have been put forward to explain the disease, including embryonic theory, migratory theory, and immunologic theories (4, 21, 22). In this case, we consider that cesarean section-related blood transmission may play a pivotal role: endometrial cells travel considerable distances from the surgical scar via the bloodstream system and finally reach the lung tissue. The absence of both pelvic and diaphragmatic endometriosis manifestations in this patient indirectly also supports the hematogenous dissemination theory (23). Notably, the extra-pelvic endometriosis almost always coexist with deep pelvic disease (1). However, it should be noted that occult pelvic lesions cannot be definitively excluded. In previous case reports, many lesions were found in the right side of the lung as in this case (24). The retrograde menstruation could explain the right-sided predominance. Peritoneal fluid containing endometrial tissue travels from the peritoneal cavity to the thorax via diaphragmatic perforations, implanting in the pleural surface and adjacent lung parenchyma (25). However, this theory does not explain all cases of TE, because not all patients have diaphragmatic defects. In addition, celomic metaplasia theory suggest that endometriosis cells can be transformed from pleural mesothelial cells (26). Chest CT is invaluable in lesion localization, with radiological findings including ground-glass opacities, pulmonary nodules, pleural effusion and pneumothorax. The periodic changes of CT lesions are favorable for the diagnosis of TE. Besides, MRI is essential for pelvic evaluation, as it can identify occult lesions and even asymptomatic diaphragmatic endometriosis (27).

Currently, there is a lack of comprehensive treatment guidelines for extra-pelvic endometriosis. Surgical intervention is the first-line treatment, which can not only remove the lesions, but also establish a clear diagnosis. The gold standard for diagnosis is histologic examination and pathological findings report endometrial glands, stroma, and hemosiderin-laden macrophages. Due to the insufficient TE samples in this case, immunohistochemistry or anti-estrogen/progesterone receptor antibodies could not be performed. Although endometriosis is considered a benign condition, there are 20% malignant transformation in extra-pelvic sites (28). However, surgical treatment does carry a certain probability of recurrence. Previous studies have reported that the AWE recurrence rate after initial surgery is 11.4%–22.2% (6, 29) and the reason may be due to residual lesions. Besides, immunological factors might also play an important role (30). In women with endometriosis, the presence of CD158a + NK cells had been found to be significantly elevated in the peritoneal fluid and peripheral blood. Interestingly, the concentration did not decrease even after surgical or drug intervention (31).

In this case, the patient chose medical intervention as an alternative treatment following a relapse of AWE. Conventional medications mainly refer to hormone therapy (combined oral contraceptives, gonadotropin-releasing hormone agonists/antagonists, and aromatase inhibitors), which works by blocking the menstrual cycle (32). Progestins, such as Dienogest, have been reported to reduce or eliminate painful symptoms in approximately 90% of endometriosis patients (33). Dienogest, an oral fourth-generation progestin, acts by binding to the progesterone receptors in endometriotic lesions, thereby inhibiting the growth and proliferation of endometriotic cells (34). Additionally, it reduces the endogenous production of estradiol and inhibits the activation of inflammatory cells (35). Progestins are available in various forms, including oral and intravenous medications, transdermal patches, intrauterine devices, as well as subcutaneous implants (36). Weight gain and breakthrough bleeding are the most common adverse effects (37, 38). In this case, the patient suffered slight weight gain and amenorrhea during the medication. Besides, GnRH agonists and antagonists inhibit the growth of endometriotic lesions by acting on hypothalamus-pituitary-ovary axis and lead a decrease in estradiol levels (39). Combined oral contraceptives are also effective on endometriosis by suppressing ovarian function which in turn reduces the cells stimulation of endometriotic cells (32). Given that NSAIDs reduce the release of prostaglandins, they remain a viable option for managing endometriosis-associated pain (40).

## Conclusion

The extra-pelvic endometriosis remains a serious disease that presents complex diagnostic challenges. When cyclic abdominal scar pain occurs, especially in women with a history of gynecological and obstetric surgery, AWE should be highly suspected to ensure timely treatment. Asymptomatic TE is even more difficult to identify and requires long-term follow-up and, if necessary, surgical intervention. Conventional treatment include surgical resection and/or pharmacological therapy aimed at alleviating pain, reducing recurrence, improving quality of life, and preserving fertility. Given the high recurrence rate of endometriosis, combined therapy may be necessary in some cases.

## Data Availability

The original contributions presented in this study are included in this article/supplementary material, further inquiries can be directed to the corresponding author.

## References

[1] Andres M Arcoverde F Souza C Fernandes L Abrao M Kho R. Extrapelvic endometriosis: A systematic review. *J Minim Invasive Gynecol.* (2020) 27:373–89. 10.1016/j.jmig.2019.10.004 31618674

[2] McCann M Schenk W Nassar A Maimone S. Thoracic endometriosis presenting as a catamenial hemothorax with discordant video-assisted thoracoscopic surgery. *Radiol Case Rep.* (2020) 15:1419–22. 10.1016/j.radcr.2020.05.064 32642009 PMC7334551

[3] Wang Y Wang X Liao K Luo B Luo J. The burden of endometriosis in China from 1990 to 2019. *Front Endocrinol (Lausanne).* (2022) 13:935931. 10.3389/fendo.2022.935931 36051388 PMC9424490

[4] Yang Y Zhao X Huang Y. Renal endometriosis mimicking cystic renal tumor: Case report and literature review. *Front Med (Lausanne).* (2021) 8:684474. 10.3389/fmed.2021.684474 34235162 PMC8255484

[5] Chiaffarino F Cipriani S Ricci E Mauri P Esposito G Barretta M Endometriosis and irritable bowel syndrome: A systematic review and meta-analysis. *Arch Gynecol Obstet.* (2021) 303:17–25. 10.1007/s00404-020-05797-8 32949284

[6] Marras S Pluchino N Petignat P Wenger J Ris F Buchs N Abdominal wall endometriosis: An 11-year retrospective observational cohort study. *Eur J Obstet Gynecol Reprod Biol X.* (2019) 4:100096. 10.1016/j.eurox.2019.100096 31650130 PMC6804734

[7] Naem A Andrikos A Constantin A Khamou M Andrikos D Lagana A Diaphragmatic endometriosis-a single-center retrospective analysis of the patients’ demographics, symptomatology, and long-term treatment outcomes. *J Clin Med.* (2023) 12:6455. 10.3390/jcm12206455 37892593 PMC10607902

[8] Naem A Roman H Martin D Krentel HA. Bird-eye view of diaphragmatic endometriosis: Current practices and future perspectives. *Front Med (Lausanne).* (2024) 11:1505399. 10.3389/fmed.2024.1505399 39618819 PMC11604425

[9] Nnoaham K Hummelshoj L Webster P d’Hooghe T de Cicco Nardone F de Cicco Nardone C Reprint of: Impact of endometriosis on quality of life and work productivity: A multicenter study across ten countries. *Fertil Steril.* (2019) 112:e137–52. 10.1016/j.fertnstert.2019.08.082 31623725

[10] Bontempo A Mikesell L. Patient perceptions of misdiagnosis of endometriosis: Results from an online national survey. *Diagnosis (Berl).* (2020) 7:97–106. 10.1515/dx-2019-0020 32007945

[11] Liu G Wang Y Chen Y Ren F. Malignant transformation of abdominal wall endometriosis: A systematic review of the epidemiology, diagnosis, treatment, and outcomes. *Eur J Obstet Gynecol Reprod Biol.* (2021) 264:363–7. 10.1016/j.ejogrb.2021.08.006 34391052

[12] Lopez-Soto A Sanchez-Zapata M Martinez-Cendan J Ortiz Reina S Bernal Manas C Remezal Solano M. Cutaneous endometriosis: Presentation of 33 cases and literature review. *Eur J Obstet Gynecol Reprod Biol.* (2018) 221:58–63. 10.1016/j.ejogrb.2017.11.024 29310043

[13] Tabibian N Swehli E Boyd A Umbreen A Tabibian J. Abdominal adhesions: A practical review of an often overlooked entity. *Ann Med Surg (Lond).* (2017) 15:9–13. 10.1016/j.amsu.2017.01.021 28203370 PMC5295619

[14] Cuffaro F Russo E Amedei A. Endometriosis, pain, and related psychological disorders: Unveiling the interplay among the microbiome, inflammation, and oxidative stress as a common thread. *Int J Mol Sci.* (2024) 25:6473. 10.3390/ijms25126473 38928175 PMC11203696

[15] Foley C Ayers P Lee T. Abdominal wall endometriosis. *Obstet Gynecol Clin North Am.* (2022) 49:369–80. 10.1016/j.ogc.2022.02.013 35636814

[16] Vagholkar K Vagholkar S. Abdominal wall endometrioma: A diagnostic enigma-a case report and review of the literature. *Case Rep Obstet Gynecol.* (2019) 2019:6831545. 10.1155/2019/6831545 31032131 PMC6457300

[17] Alaert J Lancelle M Timmermans M Tanos P Nisolle M Karampelas S. Malignancy in abdominal wall endometriosis: Is there a way to avoid it? A systematic review. *J Clin Med.* (2024) 13:2282. 10.3390/jcm13082282 38673556 PMC11050881

[18] Cocco G Delli Pizzi A Scioscia M Ricci V Boccatonda A Candeloro M Ultrasound imaging of abdominal wall endometriosis: A pictorial review. *Diagnostics (Basel).* (2021) 11:609. 10.3390/diagnostics11040609 33805519 PMC8065386

[19] Bourgioti C Preza O Panourgias E Chatoupis K Antoniou A Nikolaidou M Mr imaging of endometriosis: Spectrum of disease. *Diagn Interv Imaging.* (2017) 98:751–67. 10.1016/j.diii.2017.05.009 28652096

[20] Joseph J Sahn S. Thoracic endometriosis syndrome: New observations from an analysis of 110 cases. *Am J Med.* (1996) 100:164–70. 10.1016/s0002-9343(97)89454-5 8629650

[21] Lamceva J Uljanovs R Strumfa I. The main theories on the pathogenesis of endometriosis. *Int J Mol Sci.* (2023) 24:254. 10.3390/ijms24054254 36901685 PMC10001466

[22] Taylor H Kotlyar A Flores V. Endometriosis is a chronic systemic disease: Clinical challenges and novel innovations. *Lancet.* (2021) 397:839–52. 10.1016/S0140-6736(21)00389-5 33640070

[23] Samani E Mamillapalli R Li F Mutlu L Hufnagel D Krikun G Micrometastasis of endometriosis to distant organs in a murine model. *Oncotarget.* (2019) 10:2282–91. 10.18632/oncotarget.16889 31040919 PMC6481344

[24] Nikolettos K Patsouras A Kotanidou S Garmpis N Psilopatis I Garmpi A Pulmonary endometriosis: A systematic review. *J Pers Med.* (2024) 14:1085. 10.3390/jpm14111085 39590577 PMC11595740

[25] Kirschner P. Porous diaphragm syndromes. *Chest Surg Clin N Am.* (1998) 8:449–72. 10.1016/S1052-3359(25)00345-X 9619316

[26] Gruenwald P. Origin of endometriosis from the mesenchyme of the celomic walls. *Am J Obstetr Gynecol.* (1942) 44:470–4. 10.1016/s0002-9378(42)90484-8

[27] Tong A Cope A Waters T McDonald J VanBuren W. Best practices: Ultrasound versus mri in the assessment of pelvic endometriosis. *AJR Am J Roentgenol.* (2024) 223:e2431085. 10.2214/AJR.24.31085 39259005

[28] Krawczyk N Banys-Paluchowski M Schmidt D Ulrich U Fehm T. Endometriosis-associated malignancy. *Geburtshilfe Frauenheilkd.* (2016) 76:176–81. 10.1055/s-0035-1558239 26941451 PMC4771509

[29] Kim S Choi S Won S Shim S Lee N Kim M Cumulative recurrence rate and risk factors for recurrent abdominal wall endometriosis after surgical treatment in a single institution. *Yonsei Med J.* (2022) 63:446–51. 10.3349/ymj.2022.63.5.446 35512747 PMC9086694

[30] Horne A Missmer S. Pathophysiology, diagnosis, and management of endometriosis. *BMJ.* (2022) 379:e070750. 10.1136/bmj-2022-070750 36375827

[31] Maeda N Izumiya C Kusum T Masumoto T Yamashita C Yamamoto Y Killer inhibitory receptor Cd158a overexpression among natural killer cells in women with endometriosis is undiminished by laparoscopic surgery and gonadotropin releasing hormone agonist treatment. *Am J Reprod Immunol.* (2004) 51:364–72. 10.1111/j.1600-0897.2004.00170.x 15212673

[32] Vannuccini S Clemenza S Rossi M Petraglia F. Hormonal treatments for endometriosis: The endocrine background. *Rev Endocr Metab Disord.* (2022) 23:333–55. 10.1007/s11154-021-09666-w 34405378 PMC9156507

[33] Gezer A Oral E. Progestin therapy in endometriosis. *Womens Health (Lond).* (2015) 11:643–52. 10.2217/whe.15.42 26389558

[34] Liu Y Gong H Gou J Liu X Li Z. Dienogest as a maintenance treatment for endometriosis following surgery: A systematic review and meta-analysis. *Front Med (Lausanne).* (2021) 8:652505. 10.3389/fmed.2021.652505 33898487 PMC8058209

[35] Kim H Kim S Oh Y Lee S Chae H. Dienogest may reduce estradiol- and inflammatory cytokine-induced cell viability and proliferation and inhibit the pathogenesis of endometriosis: A cell culture- and mouse model-based study. *Biomedicines.* (2022) 10:2992. 10.3390/biomedicines10112992 36428561 PMC9687141

[36] Gheorghisan-Galateanu A Gheorghiu M. Hormonal therapy in women of reproductive age with endometriosis: An update. *Acta Endocrinol (Buchar).* (2019) 15:276–81. 10.4183/aeb.2019.276 31508191 PMC6711644

[37] Lee J Song J Yi K Lee S Lee D Shin J Effectiveness of dienogest for treatment of recurrent endometriosis: Multicenter data. *Reprod Sci.* (2018) 25:1515–22. 10.1177/1933719118779733 29848190

[38] Barra F Scala C Leone Roberti Maggiore U Ferrero S. Long-term administration of dienogest for the treatment of pain and intestinal symptoms in patients with rectosigmoid endometriosis. *J Clin Med.* (2020) 9:1054. 10.3390/jcm9010154 31935969 PMC7019573

[39] Clemenza S Vannuccini S Ruotolo A Capezzuoli T Petraglia F. Advances in targeting estrogen synthesis and receptors in patients with endometriosis. *Expert Opin Investig Drugs.* (2022) 31:1227–38. 10.1080/13543784.2022.2152325 36529967

[40] Brown J Crawford T Allen C Hopewell S Prentice A. Nonsteroidal anti-inflammatory drugs for pain in women with endometriosis. *Cochrane Database Syst Rev.* (2017) 1:CD004753. 10.1002/14651858.CD004753.pub4 28114727 PMC6464974

